# Joint resource optimization in spectrum sharing decode and forward relaying networks

**DOI:** 10.1371/journal.pone.0251509

**Published:** 2021-05-13

**Authors:** Dong Qin

**Affiliations:** School of Information Engineering, Nanchang University, Nanchang, China; Norfolk State University, UNITED STATES

## Abstract

This paper proposes an adaptive power allocation and subcarrier pairing algorithm for orthogonal frequency division multiplexing based decode and forward cognitive radio networks, where primary and secondary users achieve spectrum sharing in the same frequency band. The secondary network tries to maximize its sum rate while ensuring that the interference introduced to the primary network is below an acceptable level. Although similar problems have been investigated in traditional cooperative communication networks, it’s still an open issue in cognitive radio networks due to interference thresholds. The power consumed by the secondary network is not only limited by its own power peak, but also by the interference threshold of the primary user. Our proposed algorithm not only allocates power and pairs subcarriers reasonably, but also specifies the conditions under which the relaying link is superior to the direct transmission. Simulation results show that the sum rate of the proposed algorithm exceeds other methods and obtains a significant performance gain.

## Introduction

Cognitive radio, a dynamic spectrum access technology, has successfully alleviated the increasing scarcity of spectrum resources by allowing different entities to share the same licensed frequency band. This spectrum sharing has improved the efficiency of spectrum utilization and spawned many spectrum exploration technologies [1]. On the other hand, it has been proved that cooperative technology can effectively alleviate multipath transmission and long distance large scale fading against fluctuating channel quality in wireless communication system. Therefore, it is a wise choice to introduce cooperative technology into cognitive radio networks. For example, the energy harvesting was introduced into spectrum sharing mixed radio frequency and free space optical networks in [2], where a secondary user harvested energy from primary network. The outage probability of spectrum sharing decode and forward (DF) networks was derived in [3], where both transceiver hardware impairment and channel estimation errors were considered. In [4], the authors studied the bit error rate of M-ary quadrature amplitude modulation and M-ary phase shift keying in DF underlay spectrum sharing networks subject to additive white generalized Gaussian noise. In [5], a secondary user served as a relay for a primary user employing non orthogonal multiple access cooperative signal. Then the outage probability and throughput were calculated in closed form. Involving the order statistics theory in [6], the outage probability was derived in overlay bidirectional relaying networks with user selection.

The nature of spectrum sharing determines that scarce resources must be fully utilized to reduce waste. For instance, an zero forcing beam forming scheme and outdated channel state information were considered in [7] in multi-antenna spectrum sharing system, where the design of collaborative beam forming took into account the influence of interference channels between primary and secondary users. Based on the beam forming vector that nulled the interference to primary user, the asymptotic formulas of outage probability and bit error probability were obtained to analyze diversity and coding gains. As a win-win policy, the author in [8] allowed secondary users to exchange information with each other at the expense of helping the primary user with a portion of resources. Specifically, the secondary user took out a part of subcarriers to help the primary user achieve the target rate of bidirectional transmission, and exploited the remaining subcarriers to send its own messages. Optimal zero forcing beam former weights were obtained in [9, 10] for spectrum sharing amplify and forward (AF) relaying networks via a linear optimization method, which maximized the received signal-to-noise ratio (SNR) at the secondary user and nulled the inflicted interference on the primary user in a relaying phase. Then power allocation was numerically solved on the basis of the asymptotic error probability of the zero forcing beam forming. A full duplex cooperative opportunistic spectrum sharing protocol was considered in [11], where a secondary was granted to employ a fraction of subcarriers to transmit its own message helping primary users achieve the target rate. The opportunistic spectrum sharing protocol means that the secondary user used some particular subcarriers to help the primary user while sent its own signal by remaining subcarriers. An approximate formula of pairwise error probability was derived when imperfect successive interference cancellation was used at secondary users in non orthogonal multiple access cognitive radio networks [12], where a union bound was further deduced. Then power allocation coefficients were found to minimize the union bound under average and individual power constraints. The outage probability was considered when the secondary users formed a multihop network subject to cascaded Rayleigh fading channels in [13]. Then a heuristic algorithm was proposed to determine the number of secondary users acting as relay stations.

Although some previous works have also studied the optimization problem in cognitive radio cooperative communications, there are still some shortcomings. For example, some works only considered the narrow band channel situation instead of the multicarrier situation [2–4], so that the subcarrier pairing problem was omitted. Some worked only assumed the rate optimization problem in traditional cooperative communication, so the interference limitation of the primary user was ignored [14, 15]. Others works assumed that there was no direct link between a source and a destination due to obstacles or obstruction, so the source node had to use the relay station to transmit its own information [7, 9, 10]. At this time, the path selection problem did not exist, because there was only one path to choose from. In order to study the rate optimization problem in depth, we propose a joint algorithm of power allocation, path selection and subcarrier pairing, while previous works can be regarded as a special case of our algorithm and find the answer from it. For example, when the number of subcarriers is one, the scenario degenerates into a narrow band channel model; when the interference restriction is infinite or large enough, the scenario degenerates into a traditional cooperative communication model; when the channel gain of the direct link is zero or weak enough, the scenario degenerates into no direct link model.

Inspired by the above discussion, this paper proposes an optimal resource scheduling scheme in DF cognitive relay networks, where primary and secondary users share the same spectrum resources. In general, the primary users are paid users and enjoy the privilege of message conveyance while the transmission behavior of the secondary users has to be restricted by interference threshold that the primary users can tolerate. Under the dual constraints of its own power and interference threshold, the secondary network exhibits a different rate performance from the traditional cooperative communication network. For the sake of detailed description without omission, we divide the different constraint conditions into four cases: (1) total power and separate interference threshold constraints, (2) total power and total interference threshold constraints, (3) separate power and separate interference threshold constraints, (4) separate power and total interference threshold constraints. An joint optimization algorithm for power allocation and subcarrier pairing is proposed to ensure that the rate of the secondary users reaches the maximum and meets different constraint conditions. The proposed algorithm improves the utilization of spectrum resources and alleviates the dramatically tight spectrum congestion, especially for unregistered secondary users. Finally, for the convenience of searching, the notations frequently appearing in this paper are summarized in Table 1.

**Table 1:** List of notations.

Notations	Descriptions
*φ*_*i*,*j*_	Auxiliary variable for subcarrier pairing
*h*_*d*,*i*_	Channel coefficient from secondary source to secondary destination
*h*_*r*,*j*_	Channel coefficient from secondary relay to secondary destination
*h*_*s*,*i*_	Channel coefficient from secondary source to secondary relay
$h_{d,i}^{p}$	Channel coefficient from primary source to secondary destination
$h_{r,i}^{p}$	Channel coefficient from primary source to secondary relay
$h_{r,j}^{s}$	Channel coefficient from secondary relay to primary destination
$h_{s,i}^{s}$	Channel coefficient from secondary source to primary destination
*L*	Lagrangian function
λ_1_, λ_2_	Dual variable associated with power allocation
*μ*_1_, *μ*_2_	Dual variable associated with interference threshold
*N*_0_	Noise variance
*p*_*r*,*j*_	Transmission power of secondary relay
*p*_*s*,*i*_	Transmission power of secondary source
*q*_*s*,*i*_	Transmission power of primary source
*r*_*i*,*j*_	Rate of secondary network
*ρ*_*i*,*j*_	Subcarrier pairing binary variable
*P*_*R*_	Power budget at secondary relay
*P*_*S*_	Power budget at secondary source
*P*_*T*_	Total power budget
*PD*	Primary destination receiver
*PS*	Primary source transmitter
*Q*_1_	Interference threshold at the secondary source broadcasting phase
*Q*_2_	Interference threshold at the secondary relay forwarding phase
*Q*_*T*_	Total interference threshold
*SD*	Secondary destination receiver
*SR*	Secondary relay station
*SS*	Secondary source transmitter

## System model

**Fig 1:**
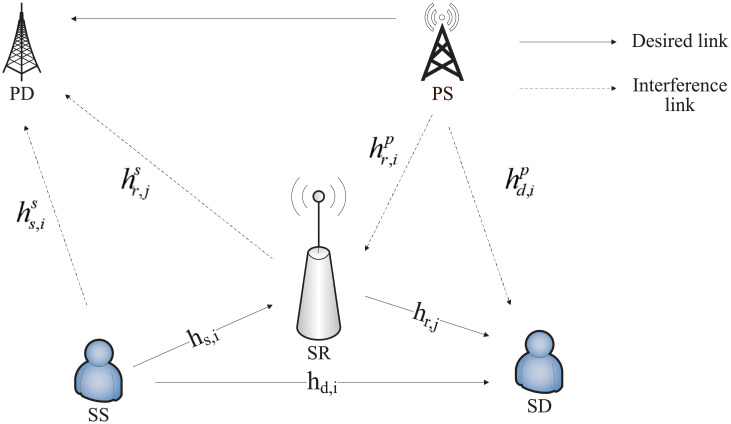
System model.

Consider a common cognitive relay network as shown in Fig 1, where a primary transmitter *PS* and a primary receiver *PD* form a primary network while a secondary transmitter *SS* delivers its message to a secondary receiver *SD* through a secondary DF relay *SR*. The primary and secondary networks use orthogonal frequency division multiplexing (OFDM) technology to share a section of licensed spectrum resources. Suppose that the channel coefficients corresponding to the transmission process from the source to the relay station *SS* → *SR*, from the relay station to the destination *SR* → *SD* and from the source to the destination *SS* → *SD* are denoted as *h*_*s*,*i*_, *h*_*r*,*i*_ and *h*_*d*,*i*_ over subcarrier *i*, respectively. The interference channel coefficients introduced by the secondary network corresponding to *SS* → *PD* and *SR* → *PD* are represented as $h_{s,i}^{s}$ and $h_{r,i}^{s}$ over subcarrier *i*, respectively. Similarly, the interference channel coefficients introduced by the primary network corresponding to *PS* → *SR* and *PS* → *SD* are $h_{r,i}^{p}$ and $h_{d,i}^{p}$, respectively. Furthermore, the transmission power of the primary transmitter is *q*_*s*,*i*_. Similarly, the transmission powers of the secondary transmitter and the relay are *p*_*s*,*i*_ and *p*_*r*,*j*_, respectively. According to the DF rule, the rate of the secondary network is given by [16, 17]

$$\begin{array}{c} r_{i,j}=\frac{1}{2}\min[\log_{2}(1+p_{s,i}a_{i}),\log_{2}(1+p_{s,i}g_{i}+p_{r,j}b_{j})] \end{array}$$ (1)

where

$$\begin{array}{c} a_{i}=\frac{{\left|h_{s,i}\right|}^{2}}{q_{s,i}{\left|h_{r,i}^{p}\right|}^{2}+N_{0}},b_{j}=\frac{{\left|h_{r,j}\right|}^{2}}{q_{s,j}{\left|h_{d,j}^{p}\right|}^{2}+N_{0}},g_{i}=\frac{{\left|h_{d,i}\right|}^{2}}{q_{s,i}{\left|h_{d,i}^{p}\right|}^{2}+N_{0}} \end{array}$$ (2)

and *N*_0_ is noise variance.

## Resource allocation under total power constraint

### Total power and separate interference constraints

In this section, we first look for the optimal resource allocation under the constraints of total power and separate interference. This problem is expressed mathematically as

$$\begin{array}{cc} \max_{\left\{p_{s,i},p_{r,j},\rho_{i,j}\right\}} & \sum_{i=1}^{N}\sum_{j=1}^{N}\rho_{i,j}r_{i,j}\\ s.t. & \sum_{i=1}^{N}\sum_{j=1}^{N}(p_{s,i}+p_{r,j})\leq P_{T}\\ & \sum_{i=1}^{N}p_{s,i}c_{i}\leq Q_{1},\sum_{j=1}^{N}p_{r,j}d_{j}\leq Q_{2}\\ & \sum_{i=1}^{N}\rho_{i,j}=1,\sum_{j=1}^{N}\rho_{i,j}=1,\forall i,j \end{array}$$ (3)

where *N* is the number of subcarrier, *P*_*T*_ is total power budget of the secondary network, *Q*_1_ and *Q*_2_ are the interference thresholds at the *SS* broadcasting phase and *SR* forwarding phase, respectively, *ρ*_*i*,*j*_ is the subcarrier pairing binary variable and $c_{i}={\left|h_{s,i}^{s}\right|}^{2}/N_{0}$, $d_{j}={\left|h_{r,j}^{s}\right|}^{2}/N_{0}$.

The Lagrangian function of problem (3) is given by

$$\begin{array}{ll} L & =\sum_{i=1}^{N}\sum_{j=1}^{N}\rho_{i,j}r_{i,j}+\lambda_{1}[P_{T}-\sum_{i=1}^{N}\sum_{j=1}^{N}\rho_{i,j}(p_{s,i}+p_{r,j})]+\mu_{1}[Q_{1}-\sum_{i=1}^{N}p_{s,i}c_{i}]+\mu_{2}[Q_{2}-\sum_{j=1}^{N}p_{r,j}d_{j}]\\ & =\sum_{i=1}^{N}\sum_{j=1}^{N}\rho_{i,j}[r_{i,j}-\lambda_{1}(p_{s,i}+p_{r,j})-\mu_{1}p_{s,i}c_{i}-\mu_{2}p_{r,j}d_{j}]+\lambda_{1}P_{T}+\mu_{1}Q_{1}+\mu_{2}Q_{2} \end{array}$$ (4)

where λ_1_, *μ*_1_ and *μ*_2_ are the dual variables associated with the power and interference constraints. In terms of the law of maximizing the minimum value, when the relay receives the same information as the destination node, the rate expressed by the minimum function in (1) reaches the maximum. Hence, substituting *p*_*r*,*j*_ = (*a*_*i*_ − *g*_*i*_)*p*_*s*,*i*_/*b*_*j*_ into (4) yields

$$L=\sum_{i=1}^{N}\sum_{j=1}^{N}\rho_{i,j}[\frac{1}{2}{\text{log}}_{2}(1+p_{s,i}a_{i})-\frac{p_{s,i}}{b_{j}}(b_{j}(\lambda_{1}+c_{i}\mu_{1})+(a_{i}-g_{i})(\lambda_{1}+d_{j}\mu_{2}))]+\lambda_{1}P_{T}+\mu_{1}Q_{1}+\mu_{2}Q_{2}$$ (5)

According to the Karush-Kuhn-Tucker (KKT) criterion [18], we derive

$$\begin{array}{c} \frac{\partial L}{\partial p_{s,i}}=\frac{a_{i}}{2\ln2(1+p_{s,i}a_{i})}-\frac{b_{j}(\lambda_{1}+c_{i}\mu_{1})+(a_{i}-g_{i})(\lambda_{1}+d_{j}\mu_{2})}{b_{j}}=0 \end{array}$$ (6)

Through (6), the power allocation of relaying link is

$$\begin{array}{c} p_{s,i}={\left[\frac{b_{j}}{2\ln2(b_{j}(\lambda_{1}+c_{i}\mu_{1})+(a_{i}-g_{i})(\lambda_{1}+d_{j}\mu_{2}))}-\frac{1}{a_{i}}\right]}^{+} \end{array}$$ (7)

$$\begin{array}{c} p_{r,j}={\left[(a_{i}-g_{i})(\frac{1}{2\ln2(b_{j}(\lambda_{1}+c_{i}\mu_{1})+(a_{i}-g_{i})(\lambda_{1}+d_{j}\mu_{2}))}-\frac{1}{a_{i}b_{j}})\right]}^{+} \end{array}$$ (8)

where (⋅)^+^ = max(0, ⋅). On the other hand, if the *SS* selects direct transmission rather than relaying link, then following a similar procedure, the power allocation is given by

$$\begin{array}{c} p_{s,i}={\left[\frac{1}{2\ln2(\lambda_{1}+c_{i}\mu_{1})}-\frac{1}{g_{i}}\right]}^{+} \end{array}$$ (9)

Next, an important question emerges: when does the secondary network choose the relaying link and when does it turn to direct transmission? Apparently, the selection criteria of the two paths will be judged by the SNR. In the relaying link, the SNR is given by

$$\begin{array}{c} \gamma_{i,j}^{r}={\left[\frac{a_{i}b_{j}}{2\ln2(b_{j}(\lambda_{1}+c_{i}\mu_{1})+(a_{i}-g_{i})(\lambda_{1}+d_{j}\mu_{2}))}-1\right]}^{+} \end{array}$$ (10)

while in the direct transmission, the SNR is given by

$$\begin{array}{c} \gamma_{i,j}^{d}={\left[\frac{g_{i}}{2\ln2(\lambda_{1}+c_{i}\mu_{1})}-1\right]}^{+} \end{array}$$ (11)

If the secondary source prefers relaying assistance, the SNR of relaying link will be greater than that of direct transmission, i.e., $\gamma_{i,j}^{r}>\gamma_{i,j}^{d}$. By comparing the SNR, the relaying mode satisfies the inequalities *a*_*i*_ > *g*_*i*_ and *b*_*j*_(λ_1_ + *c*_*i*_ *μ*_1_) > *g*_*i*_(λ_1_ + *d*_*j*_ *μ*_2_). Combining different transmission paths, the optimal power allocation is given by

$$\begin{array}{c} p_{s,i}^{⋆}=\{\begin{array}{cc} {\left[\frac{b_{j}}{2\ln2(b_{j}(\lambda_{1}+c_{i}\mu_{1})+(a_{i}-g_{i})(\lambda_{1}+d_{j}\mu_{2}))}-\frac{1}{a_{i}}\right]}^{+} & \text{if}\;a_{i}>g_{i}\;\text{and}\\ & b_{j}(\lambda_{1}+c_{i}\mu_{1})>g_{i}(\lambda_{1}+d_{j}\mu_{2})\\ {\left[\frac{1}{2\ln2(\lambda_{1}+c_{i}\mu_{1})}-\frac{1}{g_{i}}\right]}^{+} & \text{otherwise} \end{array} \end{array}$$ (12)

$$\begin{array}{c} p_{r,j}^{⋆}=\{\begin{array}{cc} {\left[\frac{a_{i}-g_{i}}{2\ln2(b_{j}(\lambda_{1}+c_{i}\mu_{1})+(a_{i}-g_{i})(\lambda_{1}+d_{j}\mu_{2}))}-\frac{a_{i}-g_{i}}{a_{i}b_{j}}\right]}^{+} & \text{if}\;a_{i}>g_{i}\;\text{and}\\ & b_{j}(\lambda_{1}+c_{i}\mu_{1})>g_{i}(\lambda_{1}+d_{j}\mu_{2})\\ 0 & \text{otherwise} \end{array} \end{array}$$ (13)

From Eqs (12) and (13), the optimal powers can be adaptively adjusted according to the captured channel information, while simple average distribution can be avoided.

Finally, we cope with the subcarrier pairing variables. Substituting the power variables *p*_*s*,*i*_ and *p*_*r*,*j*_ into (4) yields

$$\begin{array}{c} L=\sum_{i=1}^{N}\sum_{j=1}^{N}\rho_{i,j}\varphi_{i,j}+\lambda_{1}P_{T}+\mu_{1}Q_{1}+\mu_{2}Q_{2} \end{array}$$ (14)

where

$$\begin{array}{cc} \varphi_{i,j}= & \frac{1}{2}\log_{2}\left[\frac{a_{i}b_{j}}{\left(b_{j}(\lambda_{1}+c_{i}\mu_{1})+(a_{i}-g_{i})(\lambda_{1}+d_{j}\mu_{2})\right)}\right]\\ & +\frac{b_{j}(\lambda_{1}+c_{i}\mu_{1})+(a_{i}-g_{i})(\lambda_{1}+d_{j}\mu_{2})}{a_{i}b_{j}}-\frac{\log_{2}(2\ln2e)}{2} \end{array}$$ (15)

if $p_{s,i}^{⋆}>0$, *a*_*i*_ > *g*_*i*_ and *b*_*j*_(λ_1_ + *c*_*i*_ *μ*_1_) > *g*_*i*_(λ_1_ + *d*_*j*_ *μ*_2_) hold;

$$\begin{array}{c} \varphi_{i,j}=\frac{1}{2}\log_{2}(\frac{g_{i}}{\lambda_{1}+c_{i}\mu_{1}})+\frac{\lambda_{1}+c_{i}\mu_{1}}{g_{i}}-\frac{\log_{2}(2\ln2e)}{2} \end{array}$$ (16)

if $p_{s,i}^{⋆}>0$, *a*_*i*_ < *g*_*i*_ or *b*_*j*_(λ_1_ + *c*_*i*_ *μ*_1_) < *g*_*i*_(λ_1_ + *d*_*j*_ *μ*_2_) hold; and *φ*_*i*,*i*_ = 0 if $p_{s,i}^{⋆}=0$ holds. It’s easily seen that Eq (14), only binary pairing variables left, is a typical linear assignment problem in graph theory. Its optimal solution can be found in [19].

### Total power and total interference constraints

When the secondary network is constrained by total interference, this problem is expressed as

$$\begin{array}{cc} \max_{\left\{p_{s,i},p_{r,j},\rho_{i,j}\right\}} & \sum_{i=1}^{N}\sum_{j=1}^{N}\rho_{i,j}r_{i,j}\\ s.t. & \sum_{i=1}^{N}\sum_{j=1}^{N}(p_{s,i}+p_{r,j})\leq P_{T}\\ & \sum_{i=1}^{N}p_{s,i}c_{i}+\sum_{j=1}^{N}p_{r,j}d_{j}\leq Q_{T}\\ & \sum_{i=1}^{N}\rho_{i,j}=1,\sum_{j=1}^{N}\rho_{i,j}=1,\forall i,j \end{array}$$ (17)

where *Q*_*T*_ is the total interference threshold. Following a similar procedure, the optimal power allocation under the total power and total interference constraints is given by

$$\begin{array}{c} p_{s,i}^{⋆}=\{\begin{array}{cc} {\left[\frac{b_{j}}{2\ln2(b_{j}(\lambda_{1}+c_{i}\mu_{1})+(a_{i}-g_{i})(\lambda_{1}+d_{j}\mu_{1}))}-\frac{1}{a_{i}}\right]}^{+} & \text{if}\;a_{i}>g_{i}\;\text{and}\\ & b_{j}(\lambda_{1}+c_{i}\mu_{1})>g_{i}(\lambda_{1}+d_{j}\mu_{1})\\ {\left[\frac{1}{2\ln2(\lambda_{1}+c_{i}\mu_{1})}-\frac{1}{g_{i}}\right]}^{+} & \text{otherwise} \end{array} \end{array}$$ (18)

$$\begin{array}{c} p_{r,j}^{⋆}=\{\begin{array}{cc} {\left[\frac{a_{i}-g_{i}}{2\ln2(b_{j}(\lambda_{1}+c_{i}\mu_{1})+(a_{i}-g_{i})(\lambda_{1}+d_{j}\mu_{1}))}-\frac{a_{i}-g_{i}}{a_{i}b_{j}}\right]}^{+} & \text{if}\;a_{i}>g_{i}\;\text{and}\\ & b_{j}(\lambda_{1}+c_{i}\mu_{1})>g_{i}(\lambda_{1}+d_{j}\mu_{1})\\ 0 & \text{otherwise} \end{array} \end{array}$$ (19)

From (18) and (19), the path decision criterion becomes *a*_*i*_ > *g*_*i*_ and *b*_*j*_(λ_1_ + *c*_*i*_ *μ*_1_) > *g*_*i*_(λ_1_ + *d*_*j*_ *μ*_1_).

Next, the auxiliary variable associated with the subcarrier pairing is

$$\begin{array}{cc} \varphi_{i,j}= & \frac{1}{2}\log_{2}[\frac{a_{i}b_{j}}{\left(b_{j}(\lambda_{1}+c_{i}\mu_{1})+(a_{i}-g_{i})(\lambda_{1}+d_{j}\mu_{1})\right)}]\\ & +\frac{b_{j}(\lambda_{1}+c_{i}\mu_{1})+(a_{i}-g_{i})(\lambda_{1}+d_{j}\mu_{1})}{a_{i}b_{j}}-\frac{\log_{2}(2\ln2e)}{2} \end{array}$$ (20)

if $p_{s,i}^{⋆}>0$, *a*_*i*_ > *g*_*i*_ and *b*_*j*_(λ_1_ + *c*_*i*_ *μ*_1_) > *g*_*i*_(λ_1_ + *d*_*j*_ *μ*_1_) hold;

$$\begin{array}{c} \varphi_{i,j}=\frac{1}{2}\log_{2}(\frac{g_{i}}{\lambda_{1}+c_{i}\mu_{1}})+\frac{\lambda_{1}+c_{i}\mu_{1}}{g_{i}}-\frac{\log_{2}(2\ln2e)}{2} \end{array}$$ (21)

if $p_{s,i}^{⋆}>0$, *a*_*i*_ < *g*_*i*_ or *b*_*j*_(λ_1_ + *c*_*i*_ *μ*_1_) < *g*_*i*_(λ_1_ + *d*_*j*_ *μ*_1_) hold; and *φ*_*i*,*i*_ = 0 if $p_{s,i}^{⋆}=0$ holds. It is not difficult to find the optimal subcarrier pairing easily solved by using standard linear assignment method for *φ*_*i*,*j*_ in [19].

## Resource allocation under separate power constraints

### Separate power and separate interference constraints

When the secondary network is subject to separate power constraints, this problem is written as

$$\begin{array}{cc} \max_{\left\{p_{s,i},p_{r,j},\rho_{i,j}\right\}} & \sum_{i=1}^{N}\sum_{j=1}^{N}\rho_{i,j}r_{i,j}\\ s.t. & \sum_{i=1}^{N}p_{s,i}\leq P_{S},\sum_{j=1}^{N}p_{r,j}\leq P_{R}\\ & \sum_{i=1}^{N}p_{s,i}c_{i}\leq Q_{1},\sum_{j=1}^{N}p_{r,j}d_{j}\leq Q_{2}\\ & \sum_{i=1}^{N}\rho_{i,j}=1,\sum_{j=1}^{N}\rho_{i,j}=1,\forall i,j \end{array}$$ (22)

where *P*_*S*_ and *P*_*R*_ are available power budget at the secondary source *SS* and secondary relay *SR*, respectively. Similarly, the Lagrangian function of this problem (22) is given by

$$\begin{array}{ll} L & =\sum_{i=1}^{N}\sum_{j=1}^{N}\rho_{i,j}r_{i,j}+\lambda_{1}[P_{S}-\sum_{i=1}^{N}p_{s,i}]+\lambda_{2}[P_{R}-\sum_{j=1}^{N}p_{r,j}]+\mu_{1}[Q_{1}-\sum_{i=1}^{N}p_{s,i}c_{i}]+\mu_{2}[Q_{2}-\sum_{j=1}^{N}p_{r,j}d_{j}]\\ & \sum_{i=1}^{N}\sum_{j=1}^{N}\rho_{i,j}[r_{i,j}-\lambda_{1}p_{s,i}-\lambda_{2}p_{r,j}-\mu_{1}p_{s,i}c_{i}-\mu_{2}p_{r,j}d_{j}]+\lambda_{1}P_{S}+\lambda_{2}P_{R}+\mu_{1}Q_{1}+\mu_{2}Q_{2} \end{array}$$ (23)

where λ_2_ is the dual variable associating with relay power constraint. Inserting *p*_*r*,*j*_ = (*a*_*i*_ − *g*_*i*_)*p*_*s*,*i*_/*b*_*j*_ into (23) results in

$$L=\sum_{i=1}^{N}\sum_{j=1}^{N}\rho_{i,j}[\frac{1}{2}{\text{log}}_{2}(1+p_{s,i}a_{i})-\frac{p_{s,i}}{b_{j}}(b_{j}(\lambda_{1}+c_{i}\mu_{1})+(a_{i}-g_{i})(\lambda_{2}+d_{j}\mu_{2}))]+\lambda_{1}P_{T}+\mu_{1}Q_{1}+\mu_{2}Q_{2}$$ (24)

Following the similar procedures and according to the KKT rules, the optimal power allocation is given by

$$\begin{array}{c} p_{s,i}^{⋆}=\{\begin{array}{cc} {\left[\frac{b_{j}}{2\ln2(b_{j}(\lambda_{1}+c_{i}\mu_{1})+(a_{i}-g_{i})(\lambda_{2}+d_{j}\mu_{2}))}-\frac{1}{a_{i}}\right]}^{+} & \text{if}\;a_{i}>g_{i}\;\text{and}\\ & b_{j}(\lambda_{1}+c_{i}\mu_{1})>g_{i}(\lambda_{2}+d_{j}\mu_{2})\\ {\left[\frac{1}{2\ln2(\lambda_{1}+c_{i}\mu_{1})}-\frac{1}{g_{i}}\right]}^{+} & \text{otherwise} \end{array} \end{array}$$ (25)

$$\begin{array}{c} p_{r,j}^{⋆}=\{\begin{array}{cc} {\left[(a_{i}-g_{i})(\frac{1}{2\ln2(b_{j}(\lambda_{1}+c_{i}\mu_{1})+(a_{i}-g_{i})(\lambda_{2}+d_{j}\mu_{2}))}-\frac{1}{a_{i}b_{j}})\right]}^{+} & \text{if}\;a_{i}>g_{i}\;\text{and}\\ & b_{j}(\lambda_{1}+c_{i}\mu_{1})>g_{i}(\lambda_{2}+d_{j}\mu_{2})\\ 0 & \text{otherwise} \end{array} \end{array}$$ (26)

From (25) and (26), the mutual coupling relationship between channel quality and dual variables constitutes the criterion of path selection at source node. If the conditions *a*_*i*_ > *g*_*i*_ and *b*_*j*_(λ_1_ + *c*_*i*_ *μ*_1_) > *g*_*i*_(λ_2_ + *d*_*j*_ *μ*_2_) hold, the source node prefers the relaying link, otherwise direct transmission is more beneficial.

Substituting (25) and (26) into (23) to eliminate the power variables, we obtain

$$\begin{array}{c} L=\sum_{i=1}^{N}\sum_{j=1}^{N}\rho_{i,j}\varphi_{i,j}+\lambda_{1}P_{S}+\lambda_{2}P_{R}+\mu_{1}Q_{1}+\mu_{2}Q_{2} \end{array}$$ (27)

where

$$\begin{array}{cc} \varphi_{i,j}= & \frac{1}{2}\log_{2}[\frac{a_{i}b_{j}}{\left(b_{j}(\lambda_{1}+c_{i}\mu_{1})+(a_{i}-g_{i})(\lambda_{2}+d_{j}\mu_{2})\right)}]\\ & +\frac{b_{j}(\lambda_{1}+c_{i}\mu_{1})+(a_{i}-g_{i})(\lambda_{2}+d_{j}\mu_{2})}{a_{i}b_{j}}-\frac{\log_{2}(2\ln2e)}{2} \end{array}$$ (28)

if $p_{s,i}^{⋆}>0$, *a*_*i*_ > *g*_*i*_ and *b*_*j*_(λ_1_ + *c*_*i*_ *μ*_1_) > *g*_*i*_(λ_2_ + *d*_*j*_ *μ*_2_) hold;

$$\begin{array}{c} \varphi_{i,j}=\frac{1}{2}\log_{2}(\frac{g_{i}}{\lambda_{1}+c_{i}\mu_{1}})+\frac{\lambda_{1}+c_{i}\mu_{1}}{g_{i}}-\frac{\log_{2}(2\ln2e)}{2} \end{array}$$ (29)

if $p_{s,i}^{⋆}>0$, *a*_*i*_ < *g*_*i*_ or *b*_*j*_(λ_1_ + *c*_*i*_ *μ*_1_) < *g*_*i*_(λ_2_ + *d*_*j*_ *μ*_2_) hold; and *φ*_*i*,*i*_ = 0 if $p_{s,i}^{⋆}=0$ holds.

### Separate power and total interference constraints

If the secondary network is restricted to total interference constraint, this problem is written as

$$\begin{array}{cc} \max_{\left\{p_{s,i},p_{r,j},\rho_{i,j}\right\}} & \sum_{i=1}^{N}\sum_{j=1}^{N}\rho_{i,j}r_{i,j}\\ s.t. & \sum_{i=1}^{N}p_{s,i}\leq P_{S},\sum_{j=1}^{N}p_{r,j}\leq P_{R}\\ & \sum_{i=1}^{N}p_{s,i}c_{i}+\sum_{j=1}^{N}p_{r,j}d_{j}\leq Q_{T}\\ & \sum_{i=1}^{N}\rho_{i,j}=1,\sum_{j=1}^{N}\rho_{i,j}=1,\forall i,j \end{array}$$ (30)

Similarly, the optimal power allocation is given by

$$\begin{array}{c} p_{s,i}^{⋆}=\{\begin{array}{cc} {\left[\frac{b_{j}}{2\ln2(b_{j}(\lambda_{1}+c_{i}\mu_{1})+(a_{i}-g_{i})(\lambda_{2}+d_{j}\mu_{1}))}-\frac{1}{a_{i}}\right]}^{+} & \text{if}\;a_{i}>g_{i}\;\text{and}\\ & b_{j}(\lambda_{1}+c_{i}\mu_{1})>g_{i}(\lambda_{2}+d_{j}\mu_{1})\\ {\left[\frac{1}{2\ln2(\lambda_{1}+c_{i}\mu_{1})}-\frac{1}{g_{i}}\right]}^{+} & \text{otherwise} \end{array} \end{array}$$ (31)

$$\begin{array}{c} p_{r,j}^{⋆}=\{\begin{array}{cc} {\left[(a_{i}-g_{i})(\frac{1}{2\ln2(b_{j}(\lambda_{1}+c_{i}\mu_{1})+(a_{i}-g_{i})(\lambda_{2}+d_{j}\mu_{1}))}-\frac{1}{a_{i}b_{j}})\right]}^{+} & \text{if}\;a_{i}>g_{i}\;\text{and}\\ & b_{j}(\lambda_{1}+c_{i}\mu_{1})>g_{i}(\lambda_{2}+d_{j}\mu_{1})\\ 0 & \text{otherwise} \end{array} \end{array}$$ (32)

The path decision criterion becomes *a*_*i*_ > *g*_*i*_ and *b*_*j*_(λ_1_ + *c*_*i*_ *μ*_1_) > *g*_*i*_(λ_2_ + *d*_*j*_ *μ*_1_). The auxiliary variable associated with the subcarrier pairing is

$$\begin{array}{cc} \varphi_{i,j}= & \frac{1}{2}\log_{2}[\frac{a_{i}b_{j}}{\left(b_{j}(\lambda_{1}+c_{i}\mu_{1})+(a_{i}-g_{i})(\lambda_{2}+d_{j}\mu_{1})\right)}]\\ & +\frac{b_{j}(\lambda_{1}+c_{i}\mu_{1})+(a_{i}-g_{i})(\lambda_{2}+d_{j}\mu_{1})}{a_{i}b_{j}}-\frac{\log_{2}(2\ln2e)}{2} \end{array}$$ (33)

if $p_{s,i}^{⋆}>0$, *a*_*i*_ > *g*_*i*_ and *b*_*j*_(λ_1_ + *c*_*i*_ *μ*_1_) > *g*_*i*_(λ_2_ + *d*_*j*_ *μ*_1_) hold;

$$\begin{array}{c} \varphi_{i,j}=\frac{1}{2}\log_{2}(\frac{g_{i}}{\lambda_{1}+c_{i}\mu_{1}})+\frac{\lambda_{1}+c_{i}\mu_{1}}{g_{i}}-\frac{\log_{2}(2\ln2e)}{2} \end{array}$$ (34)

if $p_{s,i}^{⋆}>0$, *a*_*i*_ < *g*_*i*_ or *b*_*j*_(λ_1_ + *c*_*i*_ *μ*_1_) < *g*_*i*_(λ_2_ + *d*_*j*_ *μ*_1_) hold; and *φ*_*i*,*i*_ = 0 if $p_{s,i}^{⋆}=0$ holds.

**Fig 2:**
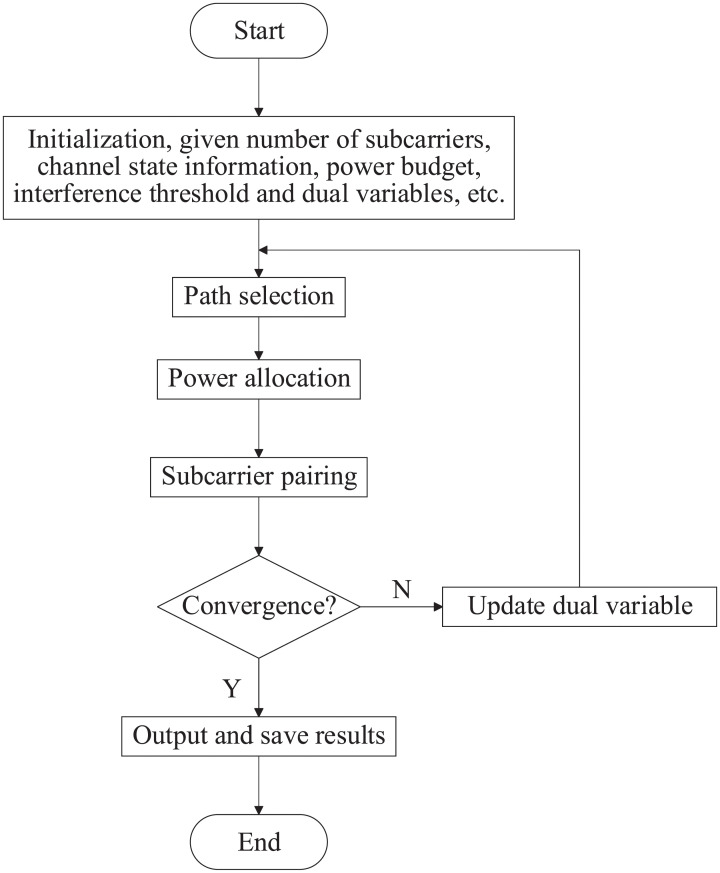
The Flowchart of the joint algorithm.

Now the analysis of four different constraint situations has been completed and a joint optimization algorithm has been proposed in this paper. Because the considered optimization problem is a mixed integer programming problem, the original problem is decomposed into subproblems with low complexity. A stepwise power allocation, path selection and subcarrier pairing strategy is adopted in terms of the dual decomposition method. First, a Lagrangian function is constructed from the objective function and constraints. Then the closed form expression of the power allocation is obtained by making the first partial derivative of the Lagrangian function zero. Further, the path selection criterion is obtained by comparing the respective benefits of the direct link and relaying transmission. Finally, substituting power variables into the Lagrangian function, subcarrier pairing is obtained through the linear assignment problem in graph theory. Through the stepwise idea and time sharing feature, continuous variables and discrete variables are treated separately to obtain a joint optimization algorithm. In order to provide readers a clearer understanding of the proposed algorithm, a flowchart is summarized in Fig 2.

It is worth noting that this paper is different from [20]. First, the cooperative protocol is different. An AF protocol is used in [20], while DF protocol is considered in this paper. In the AF protocol, the relay station does not participate in decoding the received messages, but simply amplifies and forwards them to the destination. Obviously, the advantage of this protocol lies in its simple operation and low cost. But the relay station not only amplifies the useful signal, but also enhances the noise strength. While in the DF protocol, the relay station participates in the decoding operation and then reencodes the received messages. Two different protocols determine the different behaviors of the relay station. Secondly, the research scenarios are different. There are only two scenarios in [20]: total power and separate interference constraints, separate power and separate interference constraints; while all four typical scenarios are covered in this paper. So the joint algorithm developed in this paper can be used in a wider range of scenarios than that in [20]. Third, the closed form solutions of power allocation are different. In [20], the rate formula is somewhat similar to the parallel connection of two resistors with resistance values of the channel gains from source to relay and from relay to destination; while in this paper, the rate is expressed in a minimum value form. Two different rate formulas directly produce different power allocation methods. Obviously, in the two methods, the water filling level and bowl bottom are both different. Fourth, the path selection criteria are different. In [20], path selection is related to the channel gains from the relay station to the destination and from the source to the destination. But in this paper, that is not the case. Path selection is not only related to channel gains from the relay station to the destination and from the source to the destination, but also related to the channel gain from source to relay. This is because the relay does not forward more information than it receives itself according to the principle of max flow min cut in DF protocol. Finally, the auxiliary variable *φ*_*i*,*j*_ used for subcarrier pairing is different. This is the inevitable result of different power allocation and path selection, which leads to in different subcarrier pairing results.

## Simulation results

This section compares the performance of different algorithms through Monte Carlo simulation. For ease of simulation, the relay station *SR* is located at the midpoint between the source *SS* and the destination *SD*, where the path loss exponent is 3. The number of subcarriers is chosen as *N* = 32, and the average SNR is defined as *P*_*T*_/*N*_0_. In the separate power constraint, we assume *P*_*S*_ = *P*_*R*_ = *P*_*T*_/2, and in the separate interference constraint condition, we assume *Q*_1_ = *Q*_2_ = *Q*_*T*_/2, unless otherwise specified. In order to build a more realistic simulation scenario, the channel model adopts the 12 taps typical case for urban area recommended by 3GPP TS 45.005 technical specification [21].

**Fig 3:**
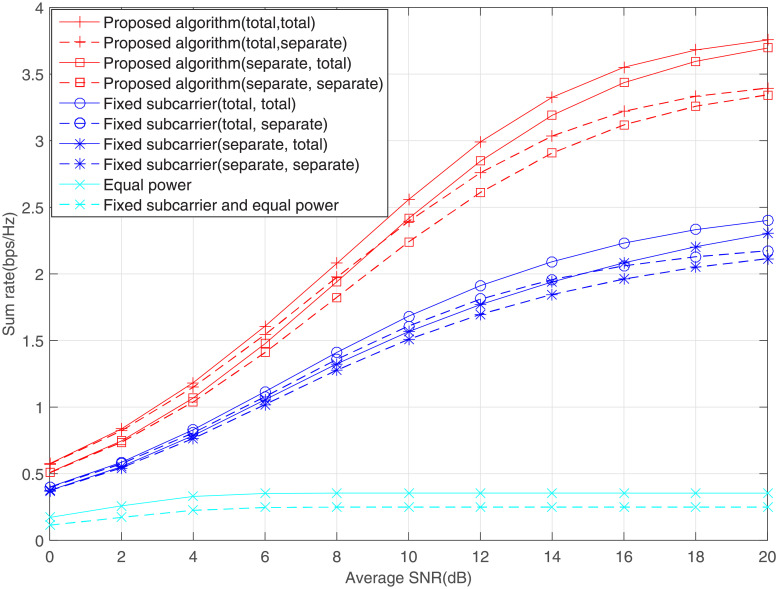
Effect of average SNR on the sum rate, where *Q*_1_ = *Q*_2_ = 0dB.

Fig 3 plots the sum rate performance of different schemes in the SNR range of 0-20 dB, where the interference threshold is attained at *Q*_1_ = *Q*_2_ = 0dB. In each figure, the first word next to each algorithm indicates power constraint, while the second word indicates interference constraint condition. For example, the first description in Fig 3 represents the performance of the proposed algorithm under the total power constraint and total interference constraint. For proposed algorithm and fixed subcarrier method, when the system suffers from the constraints of total power and total interference level, the sum rate always reaches its maximum value. An interesting phenomenon is that the sum rate of case (separate, total) is lower than that of case (total separate) at low SNR region, while the former surpasses the latter at high SNR. This interesting phenomenon fully illustrates that the power budget and interference level play different effects on the sum rate. At low SNRs, the sum rate depends on the power budget and the interference caused by the secondary users to the primary users is relatively small. However, at high SNRs, the power budget is relatively sufficient and the interference level limits the rate improvement.

**Fig 4:**
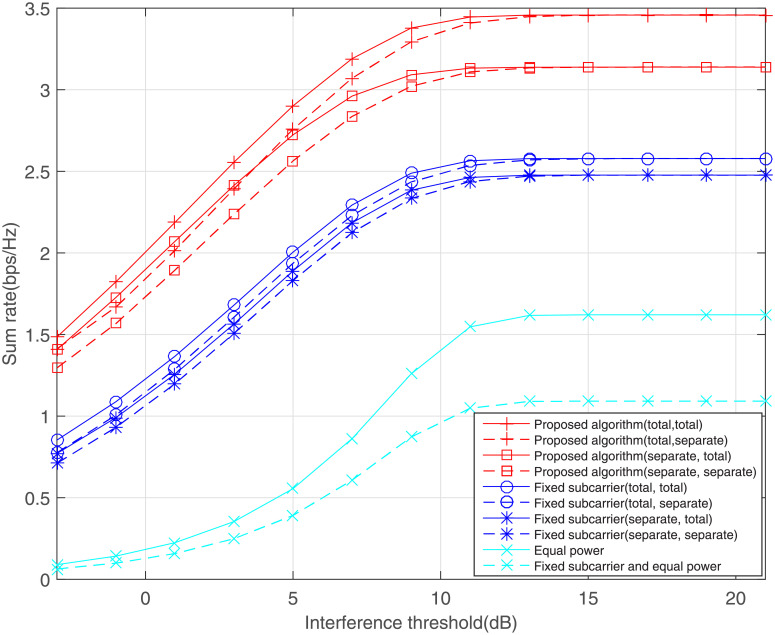
Effect of interference threshold on the sum rate, where average SNR = 10dB.

Fig 4 shows the impact of the interference threshold on the sum rate, where the power budget is maintained at average SNR = 10dB. In a departure from the rate proportional to the SNR value in traditional cooperative communications, when the SNR climbs up to a certain level, the rate will remain unchanged in cognitive radio systems. The reason is that the existence of the interference threshold limits the rate and the secondary network has to carefully adjust its transmit power so that the interference received by the primary network does not exceed the threshold. From Fig 4, the rate under total power constraint is always better than that under the separate power constraints. This is because the total power budget means that the power can be adaptively allocated between the source node and the relay station.

**Fig 5:**
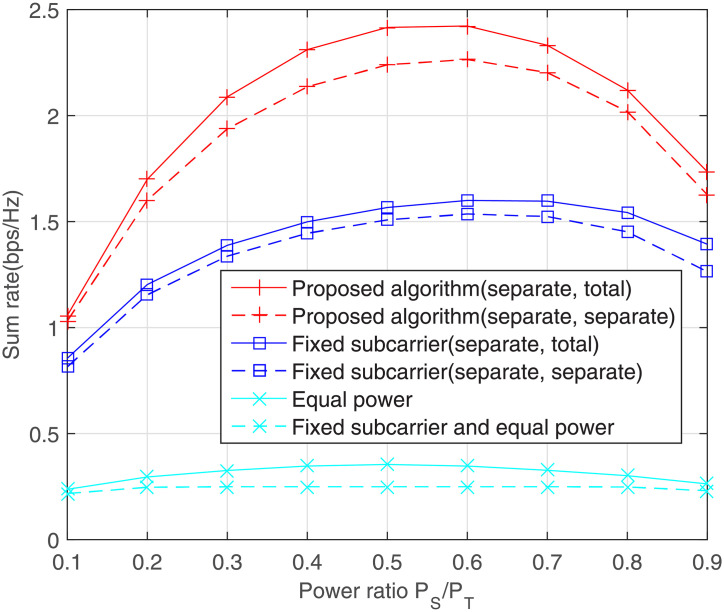
Effect of power ratio on the sum rate, where *Q*_1_ = *Q*_2_ = 0dB and average SNR = 10dB.

Fig 5 shows the impact of the relative power ratio *P*_*S*_/*P*_*T*_ on the rate. In separate constraints, it is impossible to know exactly how much power should be allocated to the source node and the relay station prior to simulation. For balanced channel conditions, the powers of the source node and the relay station are similar because it is more likely to choose relaying link than direct transmission. It can be seen from Fig 5 that the gap between the proposed algorithm and fixed subcarrier method is exactly the advantage of subcarrier pairing technology.

**Fig 6:**
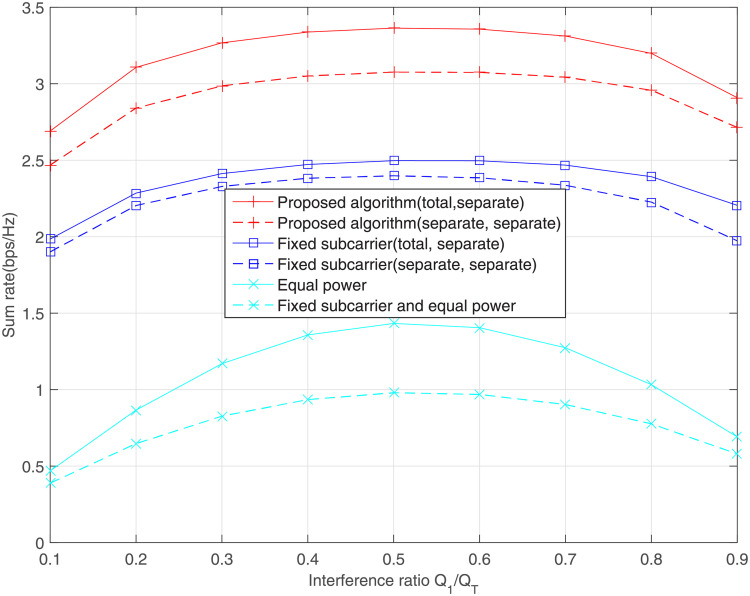
Effect of interference ratio on the sum rate, where *Q*_*T*_ = 10dB and average SNR = 10dB.

Similarly, Fig 6 illustrate the impact of relative interference ratio *Q*_1_/*Q*_*T*_ on the sum rate. Because the primary user is an authorized user and enjoys a higher priority, the interference he receives cannot exceed the minimum threshold. Under this circumstance, the sum rate of secondary users is dominated by the minimum interference threshold min(*Q*_1_, *Q*_2_). When the two interference thresholds are almost close to each other, the sum rate reaches saturation.

**Fig 7:**
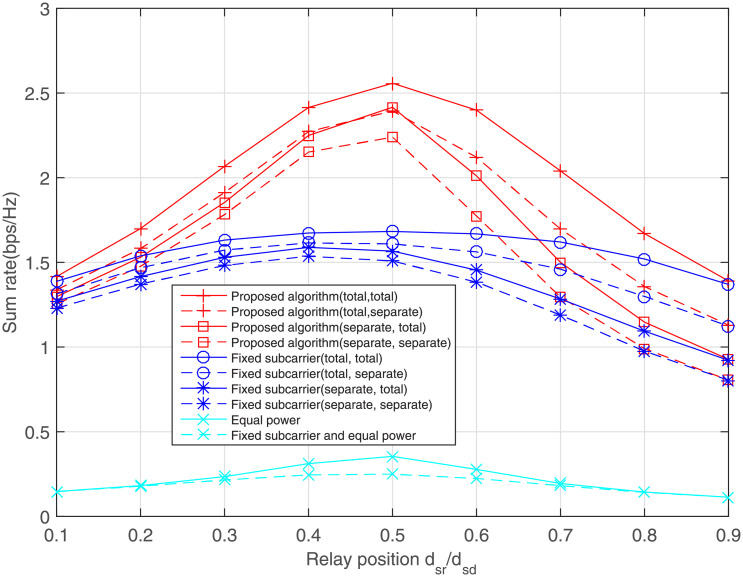
Effect of relay position on the sum rate.

The influence of relay position on sum rate is discussed in Fig 7. It can be seen from Fig 7 that for any scheme, the sum rate reaches the best performance when the relay station is located at the midpoint. This is consistent with our intuition that the relay station too close to the source or destination node will degrade the channel quality of another link. Under the same environment and parameter configuration, our proposed solution always achieves the highest rate value.

**Fig 8:**
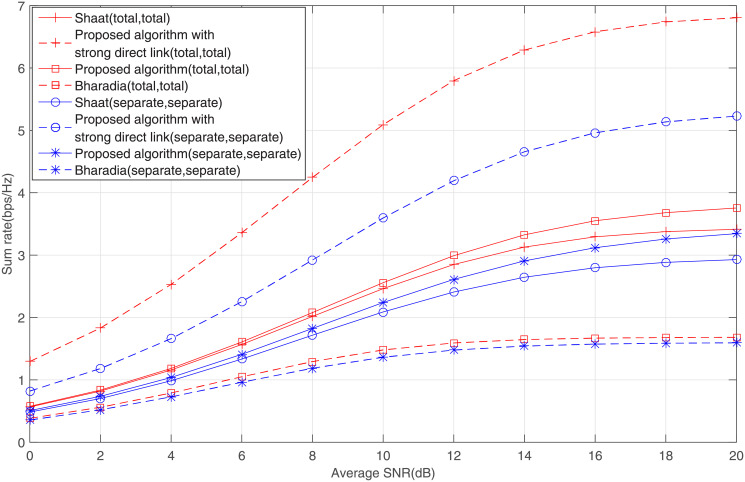
A comparison of our algorithm, Shaat’s and Bharadia’s methods.

Finally, in order to prove the superiority of the joint algorithm, we have added a comparison figure with Shaat’s [22] and Bharadia’s methods [23] in Fig 8, where the strong direct link means that the channel gain of the direct link is 10dB larger than that of the normal direct link. Note that both Shaat’s and Bharadia’s methods did not consider direct link. They attributed the absence of direct link to obstacles and long distances, so there was no path selection problem. The question of when to cooperative is actually hidden or avoided. However, in the fluctuating channel conditions, relaying transmission is not always a good choice. In this paper, direct link is incorporated and coexists with relaying transmission. In the joint algorithm, the source transmitter switches between the direct link and relaying transmission depending on the path selection criterion while maintaining the maximum sum rate performance. It can be seen from Fig 8 that when the channel quality of the direct link is good, there is obviously a large rate gap between the joint algorithm and Shaat’s and Bharadia’s. Furthermore, only a specific scenario was considered in [22, 23], either total power or separate power. But all four typical scenarios are included in this paper.

## Conclusion

This paper has discussed the rate optimization problem of spectrum sharing DF networks in detail. To solve this problem, a joint algorithm of power allocation and subcarrier pairing has been proposed. Four different constraints are classified and discussed: total power and separate interference threshold constraints, total power and total interference threshold constraints, separate power and separate interference threshold constraints, separate power and total interference threshold constraints. Simulation results show that the rate in the total constraints always exceeds that in the corresponding separate constraints.
